# ROS and calcium oscillations are required for polarized root hair growth

**DOI:** 10.1080/15592324.2022.2106410

**Published:** 2022-08-07

**Authors:** Xinxin Zhang, Ang Bian, Teng Li, Lifei Ren, Li Li, Yuan Su, Qun Zhang

**Affiliations:** a College of Life Sciences, State Key Laboratory of Crop Genetics and Germplasm Enhancement, Nanjing Agricultural University, Nanjing, P.R. China; b State Key Laboratory of Systematic and Evolutionary Botany, Institute of Botany, the Chinese Academy of Sciences, Beijing, P.R. China; c College of Computer Science, Sichuan University, Chengdu, P.R. China; d State Key Laboratory of Vegetation and Environmental Change, Institute of Botany, The Chinese Academy of Sciences, Beijing, P.R. China; e Jiangsu Key Laboratory for the Research and Utilization of Plant Resources, Institute of Botany, Jiangsu Province and Chinese Academy of Sciences (Nanjing Botanical Garden Mem. Sun Yat-Sen), Nanjing, P.R. China; f College of Life Science and Technology, Guangxi University, Nanning, P.R. China

**Keywords:** ROS, calcium, CNGC, homeostasis, RBOH, root hair

## Abstract

Root hairs are filamentous extensions from epidermis of plant roots with growth limited to the apical dome. Cell expansion undergoes tightly regulated processes, including the coordination between cell wall loosening and cell wall crosslinking, to form the final shape and size. Tip-focused gradients and oscillations of reactive oxygen species (ROS) together with calcium ions (Ca^2+^) as indispensable regulated mechanisms control rapid and polarized elongation of root hair cells. ROS homeostasis mediated by plasma membrane-localized NADPH oxidases, known as respiratory burst oxidase homologues (RBOHs), and class III cell wall peroxidases (PRXs), modulates cell wall properties during cell expansion. The expression levels of *RBOHC*, an NADPH oxidase that produces ROS, and class III *PRXs* are directly upregulated by *ROOT HAIR DEFECTIVE SIX-LIKE 4* (*RSL4*), encoding a basic-helix-loop-helix (bHLH) transcription factor, to modulate root hair elongation. Cyclic nucleotide-gated channels (CNGCs), as central regulators of Ca^2+^ oscillations, also regulate root hair extension. Here, we review how the gradients and oscillations of Ca^2+^ and ROS interact to promote the expansion of root hair cells.

## 1. Introduction

**Figure 1.**
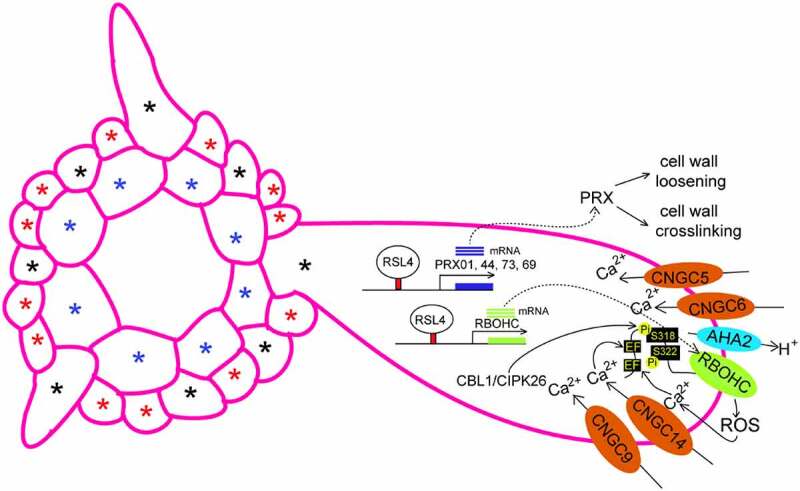
Regulation of tip growth of root hairs by ROS and Ca^2+^. This model is based on the previous works.^1,6–10^Apoplastic ROS generated by PRXs and RBOHC (RHD2) control cell wall expansion or crosslinking in the apical zone of root hairs. Ca^2+^ oscillations focused in root hair tip are modulated by CNGC5, CNGC6, CNGC9 and CNGC14. The CBL1/CIPK26 complex phosphorylates and activates RBOHC to generate ROS. RSL4 directly regulates the expression of genes encoding RBOHC and PRX01 (At1g05240), PRX44 (At4g26010), PRX73 (At5g67400), and PRX69 (AT5G64100). Blue asterisks indicate cortex. Black asterisks indicate H cell position. Red asterisks indicate N cell position.

Root hairs are unicellular extension from root epidermal cells, aiding plants in nutrient acquisition and anchorage as well as interaction with microbe in soil. The expansion rate of root hairs is determined by a complex process including the driving force from vacuolar turgor pressure, the maintenance from the exocytosis of new cell wall materials and local loosening of the existing cell wall, and exceeds 1 µm/min in *Arabidopsis*^1^. *Arabidopsis* root hairs can reach 1 mm or longer in length and approximately 10 µm in diameter.^1^ The root maturation zone is characterized by certain epidermal cells becoming root hairs. Immature epidermal cells becoming root hair or non-hair cells can be controlled by a position-dependent mechanism.^2–5^ The cortex of *Arabidopsis* primary root consists of a ring of eight cells (Figure 1). At an early stage, the epidermal cells at the junction of two underlying cortical cells (“H” position) will develop root hairs (trichoblasts), whereas non-hair cells (atrichoblasts) contact only one cortical cell (“N” position) (Figure 1). Therefore, eight immature root-hair cells in *Arabidopsis* are separated by either one or two immature non-hair cells^4,5,11^ (Figure 1). Additionally, cell-cell communication is important for establishing cell identity in the root epidermis. The Myb-like protein CAPRICE (CPC) translocates from atrichoblasts to trichoblasts and represses the expression of the negative regulator *GLABRA2* (*GL2*), thereby establishing hair cell identity.^12,13^ *ROOT HAIR DEFECTIVE6* (*AtRHD6*) encodes a basic-helix-loop-helix (bHLH) transcription factor and positively regulates the development of H cells.^14^ GL2, as an HD-Zip transcription factor, inhibits *RHD6* expression in atrichoblasts, thereby preventing differentiation into hair cells.^15–17^ The expression of *AtRHD6* was not observed in the *cpc* mutant.^18,19^ Epidermal cell differentiation can be acquired at an early stage prior to the initiation of cell elongation.^5^ The morphology of trichoblasts is distinguished from atrichoblasts prior to hair initiation.^5^ Trichoblasts in the meristematic zone display a high rate of cell division,^15^ reduced cell length before root hair initiation, relatively dense cytoplasm,^4,5^ and less vacuolated.^5^

*ROOT HAIR DEFECTIVE SIX-LIKE 4* (*RSL4*) encodes a bHLH transcription factor that promotes root hair cell elongation.^19^ The Rho-of-plant (ROP) guanosine triphosphatases (GTPases) are specifically localized to future sites of root hair emergence, and overexpression of *ROP2* results in additional and misplaced root hairs.^20–22^ Plant RAPID ALKALINIZATION FACTORS (RALFs) are secreted peptides that bind to *Catharanthus roseus* RECEPTOR-LIKE KINASE 1-like (CrRLK1L) family members such as FERONIA (FER), regulating plant cell size and shape.^23–26^ The RALF1-FER complex promotes the translation of *RSL4* and *ROP2* mRNAs.^27,28^ In turn, high-level accumulation of RSL4 protein suppresses *RALF1* expression via directly binding to the promoter of *RALF1* gene,^27,28^ fine tuning root hair elongation.

## 2. Regulation of root hair elongation by reactive oxygen species (ROS)

The cell wall, as a dynamic structure, determines the shape of root hairs. The cell wall not only provides support for expansion at the tip but also functions in resisting against the turgor pressure.^29^ Cell wall integrity is maintained through the coordination between cell wall loosening and restriction of growth via cell wall stiffening. In root hairs, cell walls are primarily composed of cellulose, xyloglucans, pectins, and hydroxyproline-rich glycoproteins that include extensins and arabinogalactan-proteins.^30,31^ Following root hair initiation, the apical zone is characterized by apoplastic ROS and cytoplasmic calcium ion (Ca^2+^) gradients. Hair cell elongation requires a balance between cell wall loosening (ROS activity) and stiffening (crosslinking of polymers). The crosslink between calcium ions and demethylated pectins forms egg-box structures, increasing cell wall stiffness.^32^ ROS can be generated in the apoplast, and have different forms with different functions, such as, dioxygen (O_2_), singlet oxygen (^1^O_2_), superoxide radical (O_2_^•−^), hydrogen peroxide (H_2_O_2_), and hydroxyl radical (OH^•^)^33^ An increase in apoplastic H_2_O_2_ concentration affects the degree of cell wall crosslinking by oxidizing cell wall compounds, thus decreasing cell expansion.^34–36^ OH^•^ mediates scission of long-chain polysaccharides, such as pectin and xyloglucan, leading to non-enzymatic loosening of cell walls.^37,38^ ROS accumulate at the apex of root hairs during elongation. In contrast, when root hairs stop growing, ROS accumulation at the apex decreases.^39,40^ Thus, abnormal accumulation of ROS in root hair cells brings about exacerbated growth or root hair burst.^6^ Respiratory burst homologs (RBOHs) and peroxidases (PRXs) are the main generators of ROS at the apoplast in plants.^41–44^ The ROS primarily generated by RBOH oxidase are required for normal root hair elongation.^34,40,45,46^ In plants, RBOHs contain six predicted membrane-spanning domains and two Ca^2+^-binding EF-hand motifs in an N-terminal cytoplasmic region, which is absent in the homologue of mammalian NOX1-4/gp91phox.^47–49^ RBOHs are activated upon Ca^2+^ binding the EF-hand and phosphorylation in the N-terminal region of RBOH.^42,50,51^ In *Arabidopsis*, root hairs are initiated correctly but incapable of elongation in *rbohc* (*root hair defective 2, rhd2*) mutants grown on medium buffered to pH 5.0.^28,34^ In maize, RHT5 (ROOTHAIRLESS 5), encoding a monocot-specific NADPH oxidase, is responsible for establishing the high levels of ROS in the tips of growing root hairs, which is required for cell elongation.^52^ Consistent with these findings, the elongation of wild-type hairs of *Arabidopsis* was inhibited by diphenyleneiodonium chloride (DPI), an inhibitor of NADPH oxidases.^40^ In addition, OH^•^ partially rescued the growth of *rbohc* mutants, but this growth lacked spatial control.^53^ Interestingly, supercentipedel1 (SCN1), a RhoGTPase GDP dissociation inhibitor (RhoGDI), can spatially restrict ROS to a single point on the trichoblast.^39^ Therefore, in *scn1* mutants, multiple tip-growing axes were formed from single bulges,^39,54^ and ROS were produced at ectopic foci in root hair cells.^39^ Trans-Golgi network-localized YPT-INTERACTING PROTEIN 4a and YPT-INTERACTING PROTEIN 4b (YIP4a/b) can induce activation and plasma membrane accumulation of ROP GTPases during root hair initiation.^22^ RHD2 accumulated at a single location in the wild type whereas it appeared at several distinct loci in the *scn1* mutant.^51^ These results suggested that SCN1 RhoGDI controls the spatial accumulation of RHD2 to regulate the ROS distribution in root hairs.^39,51^ In addition, SCN1 RhoGDI is likely to act by regulating ROP2 GTPase that is located to the hair tip, and regulates actin microfilament dynamics in root hair cells.^21,39,55^ Further study indicates that such microfilaments are required for the localization of RHD2 to the growing tip.^51^ These findings reveal that normal, long, polarized root hair growth requires not only ROS production but also correct ROS localization.

In addition to NADPH oxidase, class III PRXs can be secreted into the apoplastic space and modulate ROS levels in apoplasts to mediate root hair growth.^35^ Class III PRXs are involved in the consumption or release of H_2_O_2_ and the generation of other ROS. In the hydroxylic cycle, OH^•^ is generated from H_2_O_2_ and oxygen by Class III PRXs. In peroxidative and hydroxylic cycles, the apoplast H_2_O_2_ concentration is regulated by Class III PRXs.^35,56^ In the oxidative cycle, together with NADPH oxidase/RBOH proteins, PRXs contribute to O_2_^•−^ production by oxidizing ^1^O_2._^57^ In this regard, some PRXs appear to promote the polymerization of cell wall components, whereas others likely cause cell wall loosening via polysaccharide cleavage to promote polarized growth.^37,58,59^ Mutations in two apoplastic class III peroxidases, *PRX44* and *PRX57*, led to root hair cell wall rupture because the walls of root hair cells of the mutants were thin and mechanically weakened.^60^ Moreover, PRX62 and PRX69 stimulate *Arabidopsis* root hair elongation at low temperatures, likely through modulating ROS homeostasis and cell wall extensin-insolubilization.^6^ PRX01, PRX44, and PRX73 function in ROS homeostasis and control Tyr-crosslinking of cell wall extensins during polar expansion of root hair cells.^7,61^ RSL4 directly binds to the promoter regions of *RBOHC* and *PRX1, 44, 73*, and *69* to increase their expression, triggering apoplast oxidation and polarized root hair elongation.^6,7^

## 3. Regulation of root hair elongation by Ca^2+^

During root hair elongation Ca^2+^ is distributed primarily at the tip apex (about 1 μM) and reduced to 0.1–0.2 μM at the base of the root hair.^46,62,63^ When root hair elongation ceases, the Ca^2+^ gradient is changed.^1,46^ An increased cytoplasmic Ca^2+^ concentration provokes polar secretion, rearrangement of the actin cytoskeleton, movement of organelles, and enzyme activity,^64^ facilitating the elongation of tip-growing cells in roots.^65,66^ In addition to the Ca^2+^ concentration gradient, Ca^2+^ oscillations play a key role in transmitting signaling events for tip growth.^46,66,67^ Early studies primarily focused on the importance of the Ca^2+^ gradient and oscillations in root hair growth. Recently, Ca^2+^ channels contributing to the regulation of Ca^2+^ signatures to control polarized tip growth of root hairs have been identified.^9^ The CNGC14 nonselective cation channel mediates Ca^2+^ influx, which is required for maintaining the correct Ca^2+^ signature for the integrity of root hairs.^68^ *Cyclic nucleotide-gated channel 14* (*cngc14*) mutants exhibited very short and some branched root hairs, with altered amplitude and frequency of Ca^2+^ oscillations compared to the wild type.^68^ Moreover, CNGC14 physically interacts with calmodulin 7 (CaM7) to suppress CNGC14 activity, potentially affecting Ca^2+^ oscillations during polarized growth of root hair.^69^ Also, CNGC5, CNGC6, and CNGC9 maintain tip-focused Ca^2+^ oscillations, which are required for root hair growth and polarity. *CNGC6, CNGC9*, and *CNGC14* triple mutants showed root hair burst after transition to the rapid growth phase.^70^ Similarly, triple *cngc5/6/9* mutants exhibit markedly attenuated cytosolic Ca^2+^ oscillations in shorter and branched root hairs.^71^

An increase in Ca^2+^ concentration could impact the pH at the cell surface and ROS generation.^72^ In root hairs, apoplastic ROS, apoplastic and cytoplasmic pH, and cytoplasmic Ca^2+^ oscillations occurred with a frequency of two to four peaks per minute and lag behind the growth oscillations by 8.0, 7.0, and 5.3 s, respectively.^34,73^ RBOH proteins are activated by Ca^2+^ via direct binding of Ca^2+^ to the EF-hand motifs of RBOH proteins and Ca^2+^-induced phosphorylation of RBOH by kinases such as Ca^2+^-dependent protein kinase 5 (CPK5) and calcineurin B-interacting protein kinase (CIPK26).^10,74,75^ ROS and Ca^2+^ form a positive feedback loop in root hair elongation.^34,51,76^ ROS produced by RBOHC oxidase activate hyperpolarization-activated Ca^2+^ channels (HACCs) and stimulate Ca^2+^ influx into the cytoplasm.^40,77^ In turn, a high level of Ca^2+^ triggers RBOHC oxidase activity by binding to EF-hands and promoting the phosphorylation of the residues S318/322, maintaining active growth.^51^ Recent studies showed that calcineurin B-like protein (CBL1)-CBL-interacting protein kinase (CIPK26) complex activates RBOHC by phosphorylation, triggering ROS generation.^10^

Surface pH affects cell wall viscosity by mediating the regulation of expansins, non-hydrolytic cell wall-loosening proteins, and certain cell wall-modifying enzymes.^78–81^ Low pH promotes not only root hair initiation but also elongation. At root hair initiation sites, the pH in the apoplastic space is reduced from 5.0 to 4.5^62^. Low pH induces rapid extension of plant cell walls.^82,83^ Membrane H^+^-ATPases (AHAs) directly regulate apoplastic pH, and AHA2 is highly expressed in growing root hairs.^84,85^ FER interacts with RALF1 peptide to inhibit the activity of AHA2 possibly via Ser^899^ phosphorylation, thereby alkalizing the apoplast and suppressing cell expansion.^86,87^ Ca^2+^ modulates extracellular pH via H^+^-ATPases, which alter cell wall structure during growth.^88^ However, the root hair elongation stopped in *rbohc* mutants when exposed to pH 5.0, whereas *rbohc* mutants restored root hair elongation under pH 6.0^34^. This indicates that extracellular pH and ROS function in a coordinated and complementary manner to regulate the expansion of the growing root hair.^34^ However, little is known about the interaction of ROS and ions in modulating plant root hair development and growth.

## 4. Conclusions

Polarized root hair growth is tightly regulated by internal and external signals. ROS and Ca^2+^ are essential for the regulation of root hair elongation. Gradients and oscillations of apoplastic ROS, cytoplasmic Ca^2+^, and H^+^ are tightly linked and modulate cell wall dynamics during polar growth of root hairs. A better understanding of the mechanism underlying root hair expansion by ROS and Ca^2+^ has been achieved. However, it is unclear how ROS, Ca^2+^, and H^+^ regulate each other. Visualization of ROS and H^+^ dynamics in plant tissues and cells is challenging. Despite the identification of genes responsible for Ca^2+^ transport, further research is clearly needed to identify the Ca^2+^ transporters or channels that regulate final root hair size and to clarify how the channels are regulated by ROS. Information on the mechanism underlying root hair growth will enable the breeding of crops that can thrive under nutrient-deficient conditions and thereby increase yields.
